# “Is our choice of empirical antibiotics appropriate for patients with methicillin resistant *Staphylococcus aureus* in breast abscess?”

**Published:** 2018-12

**Authors:** Nazia Lodhi, Nadeem Khurshaidi, Rufina Soomro, Maria Saleem, Syed Sheeraz ur Rahman, Sana Anwar

**Affiliations:** Liaquat National Hospital and Medical College, Karachi, Pakistan

**Keywords:** Breast abscess, Empirical antibiotics, *Staphylococcus aureus*

## Abstract

**Background and Objectives:**

Breast abscesses remain as one of the most common reasons for females to come for a surgical consult. This retrospective cohort study includes both lactating and non-lactating females with breast abscesses. Due to changing trends in bacteriology of organisms, we need to reconsider our empirical choices of antibiotics. In our study, the main causative organism in breast abscess was *Staphylococcus aureus* with predominant species being MRSA.

**Materials and Methods:**

This is an analytical review of all breast abscesses treated in a single center from 2012 to 2015. This study included bacterial cultures, antibiotic sensitivities and resistance pattern in breast abscesses.

**Results:**

268 patients were included in the study. 143 (53.4%) were Lactational abscesses and 125 (46.6%) were non-Lactational abscesses. 169 (63.0%) harbored *S. aureus* in which 86 (50.8%) were MRSA. MRSA was the predominant organism in the Lactational group while non-Lactational group had no growth or other organisms in culture in this study. Other growing organisms were *Klebsiella pneumoniae, Bacteroides, Pseudomonas, Streptococcus* species and *Mycobacterium tuberculosis*. On comparative analysis, MRSA showed statistically a significant difference with p<0.0001, when it comes to predominant growth in lactating mothers. First line prescribed empirical antibiotics received by the patient, which is amoxicillin clavulanate, is mostly resistant. It is recommended that the institutional antibiogram targeted treatment be offered to patients with breast abscess. We also recommend ciprofloxacin with clindamycin as initial empirical therapy.

**Conclusion:**

MRSA was the most common organism seen in breast abscesses. Our first line treatment of antibiotics was resistant. Clindamycin and ciprofloxacin should be the preferred 1^st^ choice for treatment.

## INTRODUCTION

Mastitis is breast inflammation. Mastitis can become an abscess if not treated in a timely manner (1–5). Abscesses mostly occur in lactating females but can occur in non-lactating females and in all age groups. During the lactation period, incidence of mastitis is between 2% to 33% and there is 4.6% to 11% incidence of abscess formation in infected mastitis (6–9). Nipple cracks and stasis of milk due to pain are the main risk factor for abscess formation in lactating females (10–14). In non-lactating females, diabetes, obesity, smoking tobacco and black people are the main risk factors (15, 16). *Staphylococcus aureus* is the main predominant organism isolated in breast abscesses (10, 17–21). Methicillin-resistant *Staphylococcus aureus* (MRSA) infection is on the rise.

Moazzez et al. showed that 50% of cases harbor staphylococcal species in culture and 19% of all isolates from community-acquired breast abscesses were MRSA. Community acquired methicillin-resistant *S. aureus* (CA-MRSA) were mostly seen among females who lack traditional risk factors like in post-partum periods (22–24). MRSA is more common in hospital environments and in patients with indwelling medical devices and is not frequently seen in community-acquired infections. Mastitis responds poorly to routine beta-lactams and progresses to abscess formation due to delay in appropriate antibiotics. This leads to serious morbidity and interferes with continued breast feeding (5, 6, 25–28). At present, there is dearth of local literature on relationship of rising incidence of MRSA in females with breast abscesses and also on the choice of suitable antibiotics according to sensitivity. Hence, the purpose of this study was to determine the frequency of MRSA in females with breast abscesses as means to assess choice of empirical antibiotics for such a cohort.

## MATERIALS AND METHODS

This retrospective cohort study was approval by the ethics committee. Sample size was obtained from the Breast Unit of Department of General Surgery, Liaquat National Hospital in July 2016. Medical records were reviewed and data collected on a predesigned Performa for a period Jan 2012 to Dec 2015. Data included patient's age, lactational status, culture and sensitivity of the drained pus and antibiotics given. All female patients who presented with the diagnosis of breast abscess were included in this study irrespective of age and lactational status. All patients with other benign pathologies and breast cancer were excluded from the study to avoid bias. Different types of organisms were isolated in cultures but in this study we focused on patients who had MRSA in culture. Data analysis was done by SSPS version 22. Descriptive analysis of quantitative variables was done. Age is presented as median. The categorical data is presented in numbers and frequencies; for example patient lactation status & organism isolates along with their culture and sensitivities.

Patient lactational status with relation to frequent organism and sensitivity of antibiotics were determined. For inferential statistics, chi square was used as a test of significance with the p value of <0.05 as significant.

## RESULTS

A total of 268 patient's data who met the inclusion criteria in period from January 2012 to December 2015 were collected from records in Liaquat national hospital. All female patients (n= 268) met the inclusion criteria in which n=143 were lactating and n=125 were non-lactating. The age ranged from 11 to 60 years. The overall median age was 27 years. In lactating patients median age was 32 and in non-lactating patients median age was 30.

In 268 patients, 243 had unilateral breast abscesses, 6 had bilateral abscesses and 19 had axillary abscesses. In 143 lactating patients 140 were breast abscess and 3 were axillary abscesses. In 125 non-lactating patients 109 were breast and 16 were axillary abscesses. Axillary abscesses were most commonly seen in non-lactating female as appose to breast abscesses were more common in lactating females. In axillary abscesses most had no growth on final culture, 2 were *Mycobacterium tuberculosis*, 1 was *Pseudomonas aeruginosa* and 1 was *S. aureus*.

The predominant organism isolated was *Staphylococcus* n=169 (60%). Among them n=86 (50.8%) were MRSA. Other growing organisms were *Klebsiella pneumoniae, Bacteroides, Pseudomonas aeruginosa, Streptococcus* species and *Mycobacterium tuberculosis* as shown in Table 1.

**Table 1.** Isolated organisms based on culture report

**Isolated Organism**	**Count**	**percentage**
MRSA	84	31.3
MSSA	3	31
No Growth	76	28.4
*Streptococcus*	5	1.9
*Mycobacterium tuberculosis*	5	1.9
*Klebsiella pneumoniae*	4	1.5
*Pseudomonas*	3	1.1
*Bacteroides*	2	0.7
*E. coli*	2	0.7
MRSA + *Pseudomonas aeruginosa*	1	0.4
*Propionobacterium* species	1	0.4
*Staphylococcus aureua* (MRSA) + *Klebsiella*	1	0.4
*Streptococcus milleri*	1	0.4
Total	268	100

Out of 268 patients, 143 (53.4%) were lactational abscesses and 125 (46.6%) were non-lactational abscesses. In lactating patients, most common organisms found was MRSA (n=66), MSSA (n=49) and no growth in (n=23) patients. In non-lactating patients (n=125), 53 patients had no growth, 33 patients had MSSA and MRSA was found in 20 patients.

Both groups of the patients were compared for isolated organisms using chi square as a test of significance. Both group showed statistically significance difference in the organisms isolated. Details of the same are shown in Table 2.

**Table 2.** Comparison of culture isolates between the lactating and the non-lactating groups.

**Lactational status**	**Organism Isolates**	**Number of organism Isolated**	**Percentage**	**P-value**
Lactational	*Klebsiella pneumoniae*	1	0.7	
	MRSA	66	46.2	
	MSSA	49	34.3	
	No growth	23	16.1	
	*Propionobacterium* species	1	0.7	
	*Pseudomonas aeruginosa*	1	0.7	
	*Streptococcus Milleri*	1	0.7	
	*Streptococcus*	1	0.7	
	Total	143	100.0	
Non-Lactational	*Bacteroides*	2	1.6	
	*E. coli*	2	1.6	P<0.0001
	*Klebsiella pneumoniae*	3	2.4	
	MRSA	18	14.4	
	MRSA + *Pseudomonas aeruginosa*	1	0.8	
	MSSA	33	26.4	
	No Gorowth	53	42.4	
	*Pseudomonas aeruginosa*	2	1.6	
	*Staphylococcus aureus* (MRSA) + *Klebsiella pneumoniae*	1	0.8	
	*Staphylococcus aureus*	1	0.8	
	*Streptococcus*	4	3.2	
	*Mycobacterium tuberculosis*	5	4.0	
	Total	125	100.0	

Sensitivity to augmentin (amoxicillin clavulanate) is checked in all cases as cloxacillin, indicates sensitivity to all beta lactam and beta lactam inhibitor combinations. Thus cloxacillin sensitive (MSSA) means sensitive to Augmentin and cloxacillin resistant (MRSA) means resistant to augmentin. In most patients empirical antibiotics prescribed was augmentin (Amoxicillin Clavulanate) before culture and later changed as per the clinical condition and cultures because most of cultures showed *S. aureus* MRSA which was resistant to augmentin.

Out of 268 patients, 168 patients had *Staphylococci*. Out of which 3 had MSSA, 84 had MRSA and 2 had MRSA with *P. aeruginosa* and *K. pneumoniae*. In case of MSSA, the preferred choice of empiric antibiotics based on the antibiogram showed good sensitivity with cloxacillin, clindamycin, ciprofloxacin, vancomycin, fuscidic acid and amikacin. However in case of MRSA, clindamycin, ciprofloxacin, vancomycin, fuscidic acid and amikacin had good sensitivities. Details of the same are shown in Table 3.

**Table 3.** Comparison of isolated *Staphylococcus aureus* with antibiotics.

	**Cloxacillin**		**Clindamycin**		**Vancomycin**		**Ciprofloxacin**		**Fuscidic acid**		**Amikacin**	
Organism	S	R	S	R	S	R	S	R	S	R	S	R
MSSA (82)	82	0	82	0	82	0	80	2	80	2	82	0
MRSA (84)	0	84	79	5	84	0	83	1	79	5	83	1
MRSA + *Psuedomnas aeruginosa* (1)	0	1	0	1	1	0	1	0	1	0	1	0
MRSA + *Klebsiella pneumoniae* (1)	0	1	1	0	1	0	1	0	1	0	1	0

## DISCUSSION

*Staphylococcus aureus* is known to be the most common pathogenic organism in breast abscesses, but here has been scarce data on microbiologic features of breast abscesses especially in Pakistan. In most of the patients presenting with breast abscesses, both in lactational and non-lactational groups, empirically given broad spectrum antibiotics includes Gram positive coverage where the penicillin group like amoxicillin clavulanate remains the treatment of choice (4, 10, 19). In patients with co-morbidities like diabetes or history of allergies and smokers, choice of empiric antibiotics is mostly ciprofloxacin and clindamycin (17–19).

The abuse of commonly available antibiotics has led to the emergence of resistant organisms and perhaps the reason behind the resistance comes from community acquired cultures. Hence, we are prescribing empirical antibiotics which are most likely to be resistant in the very first place.

In 2008, Stafford and colleagues (29) found that the most common pathogen in clinically significant puerperal breast abscess in their population was community-acquired MRSA (67%, n=18). Their case number was small and their data came from women who required hospitalization over a 9-years span. Thomsen and colleagues (30) emphasized breast abscess drainage which increased favorable outcome by 50% and significantly decreased the duration of symptoms. In 96% of cases, appropriate antibiotics further reduced the persistent symptoms.

Walker et al. (12) showed that anaerobic and mix organisms more commonly seen in non-lactating females and in smokers and those who had recurrent abscess. In these patients, appropriate broad spectrum antibiotics started from the very beginning had beneficial effects. Our review of literature included studies from 1993 to 2015 to incorporate changing trends in MRSA development. The frequency of reported MRSA has ranged from 7% to 100% in the community setup in patients with breast abscess with a mean of around 51.8%. Though our study period covered 4 years from 2012 to 2015, our findings also corroborate the same mean percentage of 51% as per the literature search.

Etiology of rise in MRSA is not known. However it can be hypothesized that these lactating mothers might have had some hospital exposure that during their peripartum period could have led to emergence of MRSA. Further studies / prospective trials are needed to justify such an argument (31–34).

In our study MRSA is the main organism. All MRSA were susceptible to ciprofloxacin, clindamycin, vancomycin, fuscidic acid and amikacin. Based on this premise, one can assume that the likely organism isolated will be MRSA which is likely going to be resistant to augmentin (amoxicillin clavulanate) as evident from our data. Hence plea for starting clindamycin or ciporfloxacin as first line or empiric treatment in patients with breast abscess can be made. Also clindamycin has a good anaerobic coverage hence would also cover the *Bacteroides*. Vancomycin is a treatment of choice either in MRSA or in patients with signs of systemic infection. For a simple breast abscess where outpatient oral therapy is warranted, choice of clindamycin and or ciprofloxacin is pretty reasonable. Also *Clostridium difficile* is not a problem for developing countries yet.

Our study is strengthened by the fact that it has the largest sample size reported so far. Since our centre is the largest referral centre in the region for breast disease, collected data can be considered as a good representation of the true community sample to make necessary inferences. Our study is limited by its retrospective design, lack of possible incomplete history regarding previous exposure and any intervention prior to hospital visit. Multicenter trials or collaborative data from the region can help us in validating our results. Also we lack facilities to confirm the source of MRSA whether its hospital or community acquired through testing by Pulsed-field Gel electrophoresis (PFGE) or testing for Panton Valentine Leukocidin (PVL). Some of the assumptions therefore would have limited inferences (35–37).

In conclusion, MRSA is the most common organism seen in breast abscesses. Our first line treatment of antibiotics is likely to be resistant. Clindamycin and ciprofloxacin should be the preferred 1^st^ choice of treatment.
